# Supplementary material for: Interleukin-18 Is a Prognostic Biomarker Correlated with CD8+ T Cell and Natural Killer Cell Infiltration in Skin Cutaneous Melanoma
Source: J Clin Med. 2019 Nov 15;8(11):1993. doi: 10.3390/jcm8111993 (PMC6912818; doi:10.3390/jcm8111993)
Supplement: Supplementary file 1 [file jcm-08-01993-s001.pdf]

Supplementary Table S1. Tumor Abbreviations.

Abbreviation	Type of Cancer
ACC	Adrenocortical carcinoma
BLCA	Bladder Urothelial Carcinoma
BRCA	Breast invasive carcinoma
CESC	Cervical squamous cell carcinoma and endocervical adenocarcinoma
CHOL	Cholangio carcinoma
COAD	Colon adenocarcinoma
DLBC	Lymphoid Neoplasm Diffuse Large B-cell Lymphoma
ESCA	Esophageal carcinoma
GBM	Glioblastoma multiforme
HNSC	Head and Neck squamous cell carcinoma
KICH	Kidney Chromophobe
KIRC	Kidney renal clear cell carcinoma
KIRP	Kidney renal papillary cell carcinoma
LAML	Acute Myeloid Leukemia
LGG	Brain Lower Grade Glioma
LIHC	Liver hepatocellular carcinoma
LUAD	Lung adenocarcinoma
LUSC	Lung squamous cell carcinoma
MESO	Mesothelioma
OV	Ovarian serous cystadenocarcinoma
PAAD	Pancreatic adenocarcinoma
PCPG	Pheochromocytoma and Paraganglioma
PRAD	Prostate adenocarcinoma
READ	Rectum adenocarcinoma
SARC	Sarcoma
SKCM	Skin Cutaneous Melanoma
STAD	Stomach adenocarcinoma
TGCT	Testicular Germ Cell Tumors
THCA	Thyroid carcinoma
THYM	Thymoma
UCEC	Uterine Corpus Endometrial Carcinoma
UCS	Uterine Carcinosarcoma
UVM	Uveal Melanoma

Supplementary Table S2. Cox regression results for *IL18* with TCGA data in various kinds of cancers by OncoLnc (**http://www.oncolnc.org/**). The data are arranged in ascending order of the *P*-value.

Cancer	Cox Coefficient	*P*-value	KDR-Corrected	Median Expression	Mean Expression
LGG	0.471	6.30×10^{-7}	8.60×10^{-6}	135.92	199.61
SKCM	–0.256	1.60×10^{-4}	3.78×10^{-3}	87.61	168.84
SARC	–0.355	1.00×10^{-3}	3.23×10^{-2}	99.82	250.87
PAAD	0.294	7.70×10^{-3}	6.79×10^{-2}	779.42	822.9
BRCA	–0.186	3.00×10^{-2}	3.18×10^{-1}	150.97	213.42
COAD	–0.137	2.00×10^{-1}	6.12×10^{-1}	427.25	506.62
GBM	0.095	2.70×10^{-1}	8.73×10^{-1}	306.02	371.7
LUAD	0.061	4.20×10^{-1}	6.52×10^{-1}	374.46	461.64
STAD	–0.061	4.80×10^{-1}	8.05×10^{-1}	502.09	632.43
BLCA	–0.055	4.80×10^{-1}	7.15×10^{-1}	320.41	433.14
CESC	–0.088	5.00×10^{-1}	7.93×10^{-1}	451.44	620.3
LAML	0.076	5.00×10^{-1}	8.07×10^{-1}	786.95	847.65
KIRP	0.09	5.70×10^{-1}	7.29×10^{-1}	661.05	790.37
OV	–0.029	6.90×10^{-1}	9.43×10^{-1}	240.56	324.53
UCEC	–0.039	7.00×10^{-1}	9.95×10^{-1}	180.52	267.72
READ	–0.07	7.10×10^{-1}	9.81×10^{-1}	342.93	415.37
LIHC	0.028	7.60×10^{-1}	8.95×10^{-1}	77.52	152.55
KIRC	0.011	9.00×10^{-1}	9.33×10^{-1}	405.52	518.67
HNSC	0.005	9.40×10^{-1}	9.79×10^{-1}	719.41	880.93
ESCA	0.007	9.50×10^{-1}	9.94×10^{-1}	550.73	691.93
LUSC	0	1.00×10^{0}	1.00×10^{-0}	356.82	433.15

Supplementary Table S3. Datasets of *IL18* expression in SKCM (Oncomine database).

Dataset	Normal (Cases)	Tumor (Cases)	Fold change	*t*-Test	*P*-value	Rank (%)
Riker	Skin (4)	Cutaneous Melanoma (14)	–6.885	–3.822	7.80×10^{-4}	6
Talantov	Skin (7)	Cutaneous Melanoma (45)	–3.044	–2.426	0.021	29

Supplementary Table S4. GEO Datasets used in Figure 3.

Figure	GEO Dataset	Cases	Type	Platform	Ref (PMID)
Figure3a	GSE7553	87	Expression profiling by array Platforms	GPL570	18442402
Figure3b,c	GSE19234	44	Expression profiling by array Platforms	GPL570	19915147

Supplementary Table S5. Correlation constants and *p*-values in Figure 4.

Cancer	Variable	Partial.cor	*p*-value
SKCM	Purity	–0.67406	5.73×10^{-62}
	B Cell	0.201717	1.69×10^{-5}
	CD8+ T Cell	0.463666	9.90×10^{-25}
	CD4+ T Cell	0.264977	1.33×10^{-8}
	Macrophage	0.383875	2.36×10^{-17}
	Neutrophil	0.50585	9.72×10^{-31}
	Dendritic Cell	0.532757	4.60×10^{-34}
SARC	Purity	–0.53317	2.13×10^{-19}
	B Cell	0.333087	1.34×10^{-7}
	CD8+ T Cell	0.239157	0.000184
	CD4+ T Cell	0.684043	2.55×10^{-34}
	Macrophage	0.632313	1.21×10^{-27}
	Neutrophil	0.493529	2.92×10^{-16}
	Dendritic Cell	0.708182	5.36×10^{-38}
BRCA	Purity	–0.37205	5.05×10^{-34}
	B Cell	0.367116	1.55×10^{-32}
	CD8+ T Cell	0.217488	6.50×10^{-12}
	CD4+ T Cell	0.368832	2.28×10^{-32}
	Macrophage	0.130851	3.87×10^{-5}
	Neutrophil	0.390455	5.40×10^{-36}
	Dendritic Cell	0.440186	2.42×10^{-46}
COAD	Purity	–0.15924	0.001268
	B Cell	0.205531	3.14×10^{-5}
	CD8+ T Cell	0.220389	7.39×10^{-6}
	CD4+ T Cell	–0.15222	0.002212
	Macrophage	–0.13295	0.007453
	Neutrophil	0.076431	0.126517
	Dendritic Cell	0.067143	0.1791

Supplementary Table S6. Correlation analysis between *IL18* and relate genes and markers of immune cells in TIMER.

Description	Gene markers	SARC				BRCA			
		None		Purity		None		Purity	
		Cor	*P*	Cor	*P*	Cor	*P*	Cor	*P*
**CD8+ T cell**	*CD8A*	0.644	***	0.565	***	0.508	***	0.399	***
	CD8B	0.670	***	0.584	***	0.502	***	0.399	***
**T cell (general)**	*CD3D*	0.739	***	0.656	***	0.592	***	0.501	***
	CD3E	0.703	***	0.622	***	0.582	***	0.486	***
	CD2	0.722	***	0.641	***	0.596	***	0.508	***
**B cell**	*CD19*	0.566	***	0.501	***	0.469	***	0.361	***
	CD79A	0.539	***	0.407	***	0.460	***	0.341	***
**Monocyte**	*CD86*	0.867	***	0.810	***	0.603	***	0.537	***
	CD115 (CSF1R)	0.858	***	0.797	***	0.511	***	0.412	***
**TAM**	*CCL2*	0.563	***	0.461	***	0.435	***	0.338	***
	CD68	0.803	***	0.720	***	0.528	***	0.454	***
	IL10	0.718	***	0.601	***	0.446	***	0.356	***
**M1 Macrophage**	*INOS (NOS2)*	0.018	0.689	–0.127	**	0.029	0.203	0.004	0.858
	IRF5	0.816	***	0.761	***	0.397	***	0.345	***
	COX2 (PTGS2)	–0.151	**	–0.102	0.024	0.210	***	0.086	**
**M2 Macrophage**	*CD163*	0.792	***	0.703	***	0.419	***	0.335	***
	VSIG4	0.785	***	0.694	***	0.391	***	0.299	***
	MS4A4A	0.806	***	0.721	***	0.494	***	0.400	***
**Neutrophils**	*CD66b (CEACAM8)*	0.011	0.806	–0.004	0.934	0.015	0.497	0.020	0.369
	CD11b (ITGAM)	0.845	***	0.784	***	0.540	***	0.467	***
	CCR7	0.512	***	0.440	***	0.497	***	0.382	***
**Natural killer cell**	*KIR2DL1*	0.330	***	0.260	***	0.296	***	0.225	***
	KIR2DL3	0.431	***	0.347	***	0.324	***	0.248	***
	KIR2DL4	0.528	***	0.436	***	0.386	***	0.319	***
	KIR3DL1	0.371	***	0.276	***	0.294	***	0.209	***
	KIR3DL2	0.460	***	0.358	***	0.387	***	0.296	***
	KIR3DL3	0.182	***	0.124	*	0.208	***	0.162	***
	KIR2DS4	0.300	***	0.235	***	0.297	***	0.220	***
	KLRK1 (NKG2D)	0.697	***	0.516	***	0.198	**	0.138	*
	NCR1 (NKp46)	0.526	***	0.362	***	0.159	*	0.095	0.055
	NCR2 (NKp44)	0.255	***	0.179	**	0.105	0.024	0.092	0.065
	NCR3 (NKp30)	0.713	***	0.503	***	0.133	0.004	0.066	0.187
**Dendritic cell**	*HLA-DPB1*	0.787	***	0.710	***	0.593	***	0.503	***
	HLA-DQB1	0.692	***	0.593	***	0.519	***	0.435	***
	HLA-DRA	0.770	***	0.683	***	0.630	***	0.553	***
	HLA-DPA1	0.758	***	0.672	***	0.595	***	0.510	***
	BDCA-1 (CD1C)	0.339	***	0.300	***	0.415	***	0.278	***
	BDCA-4 (NRP1)	0.065	0.150	–0.092	0.042	0.136	***	–0.005	0.817
	CD11c (ITGAX)	0.653	***	0.579	***	0.579	***	0.502	***
**Th1**	*T-bet (TBX21)*	0.661	***	0.588	***	0.559	***	0.461	***
	STAT4	0.689	***	0.569	***	0.537	***	0.430	***
	STAT1	0.373	***	0.301	***	0.320	***	0.269	***
	IFN-g (IFNG)	0.539	***	0.441	***	0.510	***	0.430	***
	TNF-a (TNF)	0.393	***	0.301	***	0.360	***	0.317	***
**Th2**	*GATA3*	0.386	***	0.269	***	–0.288	***	–0.207	***
	STAT6	–0.059	0.191	–0.025	0.576	0.064	*	0.005	0.837
	STAT5A	0.532	***	0.474	***	0.246	***	0.133	***
	IL13	0.132	*	0.092	0.042	0.161	***	0.106	***
**Tfh**	*BCL6*	0.169	**	0.165	**	0.032	0.156	–0.014	0.528
	IL21	0.281	***	0.192	***	0.325	***	0.258	***
**Th17**	*STAT3*	–0.029	0.519	–0.070	0.124	–0.016	0.488	–0.075	**
	IL17A	0.019	0.682	–0.014	0.758	0.204	***	0.143	***
**γδ T cell**	*RORC (RORγt)*	0.189	*	0.182	*	–0.064	***	–0.059	0.061

	CD27	0.68	***	0.578	***	0.544	***	0.434	***
	CCR5	0.719	***	0.642	***	0.595	***	0.508	***
	CXCR6	0.697	***	0.603	***	0.560	***	0.461	***
**Treg**	*FOXP3*	0.412	***	0.275	***	0.489	***	0.403	***
	CCR8	0.431	***	0.342	***	0.403	***	0.334	***
	STAT5B	–0.253	***	–0.114	0.012	–0.070	*	–0.154	***
	TGFβ (TGFB1)	0.552	***	0.446	***	0.281	***	0.150	***
**T cell exhaustion**	*PD-1 (PDCD1)*	0.617	***	0.497	***	0.534	***	0.438	***
	CTLA4	0.609	***	0.514	***	0.560	***	0.481	***
	LAG3	0.410	***	0.337	***	0.481	***	0.430	***
	TIM-3 (HAVCR2)	0.876	***	0.823	***	0.577	***	0.508	***
	GZMB	0.654	***	0.546	***	0.517	***	0.429	***

SARC, sarcoma; BRCA, Breast invasive carcinoma.; TAM, tumor-associated macrophage; Th, T helper cell; Tfh, Follicular helper T cell; Treg, regulatory T cell; Cor, R value of Spearman's correlation; None, correlation without adjustment. Purity, correlation adjusted by purity. *$P < 0.01$; **$P < 0.001$; ***$P < 0.0001$.

Supplementary Table S7. Correlation analysis between *IL18* and relate genes of γδ T cells in SKCM and COAD (TIMER).

Description	Gene markers	SKCM				COAD			
		None		Purity		None		Purity	
		Cor	*P*	Cor	*P*	Cor	*P*	Cor	*P*
**γδ T cell**	*RORC (RORγt)*	0.398	***	0.238	***	0.062	0.184	0.061	0.220
	CD27	0.747	***	0.538	***	0.081	0.082	–0.007	0.895
	CCR5	0.805	***	0.655	***	0.047	0.317	–0.038	0.447
	CXCR6	0.772	***	0.591	***	0.18	***	0.126	*

SKCM, skin cutaneous melanoma; COAD, Colon Adenocarcinoma; Cor, R value of Spearman's correlation; None, correlation without adjustment. Purity, correlation adjusted by purity. *$P < 0.01$; **$P < 0.001$; ***$P < 0.0001$.

Supplementary Table S8. Correlation analysis between *IL18* and relate genes and markers of immune cells in GEPIA.

9	Gene markers	SARC				BRCA			
		Tumor		Normal		Tumor		Normal	
		R	*P*	R	*P*	R	*P*	R	*P*
**CD8⁺ T cells**	*CD8A*	0.55	***	N/A	N/A	0.43	***	0.42	***
	CD8B	0.57	***	N/A	N/A	0.33	*	0.36	***
**NK cells**	*KIR2DL1*	0.3	***	N/A	N/A	–0.056	0.85	–0.076	0.2
	KIR2DL3	0.31	***	N/A	N/A	0.23	***	–0.027	0.65
	KIR2DL4	0.4	***	N/A	N/A	0.27	***	–0.062	0.29
	KIR3DL1	0.19	*	N/A	N/A	0.24	***	–0.026	0.66
	KIR3DL2	0.53	***	N/A	N/A	0.24	***	0.22	**
	KIR3DL3	0.2	*	N/A	N/A	–0.017	0.57	0.029	0.63
	KIR2DS4	0.39	***	N/A	N/A	0.19	***	0.072	0.22
	KLRK1	0.31	***	N/A	N/A	0.44	***	0.079	0.18
	NCR1	0.38	***	N/A	N/A	0.39	***	0.14	0.016
	NCR2	0.066	0.29	N/A	N/A	0.089	*	0.079	0.18
	NCR3	0.53	***	N/A	N/A	0.21	***	0.41	***
**γδ T cell**	*RORC*	0.53	0.4	N/A	N/A	–0.021	0.49	0.1	0.081
	CD27	0.63	***	N/A	N/A	0.37	**	0.28	**
	CCR5	0.61	***	N/A	N/A	0.48	***	0.59	***
	CXCR6	0.58	***	N/A	N/A	0.46	***	0.44	***

*P < 0.01; **P < 0.001; ***P < 0.0001.

Supplementary Table S9. Correlation analysis between *IL18* and relate genes of γδ T cells in SKCM and COAD (GEPIA).

Cell type	Gene markers	SKCM				COAD			
		Tumor		Normal		Tumor		Normal	
		R	*P*	R	*P*	R	*P*	R	*P*
**γδ T cell**	*RORC (RORγt)*	0.11	0.016	0.37	***	–0.014	0.82	0.69	***
	CD27	0.67	***	–0.088	0.038	0.057	0.34	0.62	***
	CCR5	0.7	***	–0.11	*	0.063	0.3	–0.088	0.099
	CXCR6	0.65	***	0.87	***	0.14	0.021	0.53	***

*P < 0.01; **P < 0.001; ***P < 0.0001.
